# Myopericarditis as a Manifestation of Long COVID Syndrome

**DOI:** 10.7759/cureus.19449

**Published:** 2021-11-10

**Authors:** Olga Vera-Lastra, Abihai Lucas-Hernández, Jose E Ruiz-Montiel, Viviana R Gonzalez-Rodriguez, Luis F Pineda-Galindo

**Affiliations:** 1 Internal Medicine, Hospital de Especialidades Dr. Antonio Fraga Mouret, Instituto Mexicano del Seguro Social, Mexico City, MEX; 2 Division of Post-graduate Studies, Faculty of Medicine, Universidad Nacional Autónoma de México, Mexico City, MEX; 3 Multidisciplinary Academic Division of Comalcalco, Universidad Juárez Autónoma de Tabasco, Tabasco, MEX

**Keywords:** treatment, diagnosis, covid-19, myopericarditis, long covid

## Abstract

The main presentation of severe acute respiratory syndrome coronavirus 2 (SARS-CoV-2) infection is respiratory. However, there are extrapulmonary manifestations such as myocardial and pericardial injury. The term long COVID syndrome describes the persistence of symptoms in patients who have recovered from the infection. A 31-year-old man presented with mild coronavirus disease 2019 (COVID-19) symptoms for three days. Two weeks later, he developed chest pain, pericardial rub, and pericardial effusion; he underwent echocardiography showing pericarditis and an MRI which revealed inferoseptal hypokinesia and mild global myocardial hyperintensity, cardiac scintigraphy with Ga-67, and an inflammatory process in the myocardium. He was treated with methylprednisolone pulse (1g IV/day) and tapering prednisone (5 mg/day), with gradual evolution of symptoms for one year. In conclusion, this is a patient without comorbidities with clinical, laboratory, and imaging diagnosis of myopericarditis as a manifestation of long COVID syndrome.

## Introduction

As of October 2021, more than 230 million cases of coronavirus disease 2019 (COVID-19) have been confirmed [1]. The main clinical presentation is primarily related to the respiratory system; however, the extrapulmonary manifestations have been widely described. In the cardiovascular system, it presents as arrhythmias, myocarditis, pericarditis, heart failure, cardiogenic shock, and cardiomyopathy, contributing to higher mortality during the acute phase. There have been many cases described during this phase of the disease, but not as long-term complications [2,3]. It has been proposed that the damage to myocardial cells is mediated by the presence of angiotensin-converting enzyme 2 (ACE2) receptors, thereby facilitating the direct entry of severe acute respiratory syndrome coronavirus 2 (SARS-CoV-2) and causing cardiotoxicity. Abnormal activation of T cells, monocytes, and cytokine leads to a systemic hyperinflammatory response with reduced coronary blood flow, hence myocardial hypoxemia and micro thrombogenesis [4]. Recent studies have mentioned that it is a type 2 infarction given by the increased demand on myocardial tissue due to systemic inflammation; similarly, thrombotic events and right ventricular dilation are explained [5]. The persistence of clinical manifestations after an acute process of SARS-CoV-2 infection are denoted within the term “long COVID,” also described as post-COVID syndrome; the difference between these two terms is time of presentation [6]. This report presents the case of a young patient with fluctuating symptoms of myopericarditis after one year as part of long COVID syndrome.

## Case presentation

**Figure 1:**
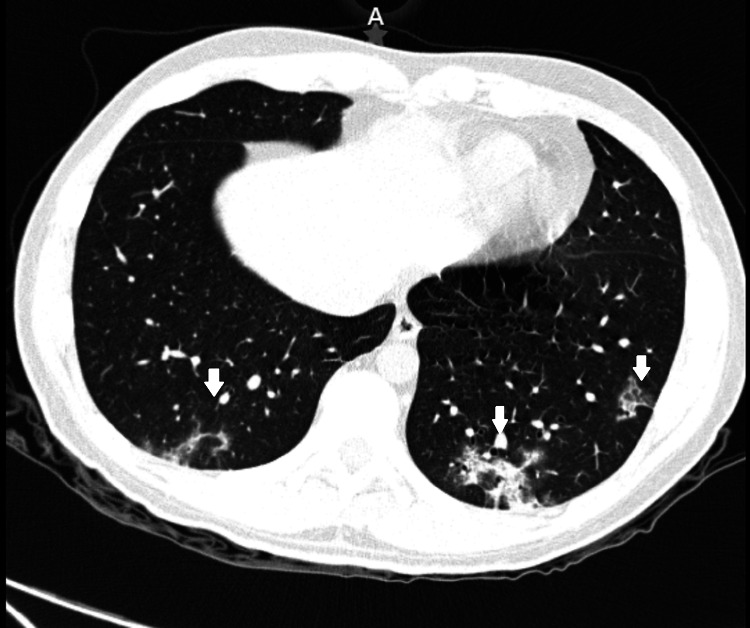
High Resolution Chest Computer Tomography Basal ground-glass opacities

We present the case of a 31-year-old male healthcare worker with no cardiovascular risk history. He was diagnosed in May 2020 with COVID-19 by RT-PCR test, presenting mild manifestations (fever, myalgias, arthralgias), initially treated as an outpatient. Two weeks later, he developed severe chest pain, radiating to the scapular region, predominantly in the evening, an electrocardiogram was taken where no signs of infarction were seen. A chest tomography was performed and showed a few patchy glass opacities (Figure 1), therefore he was hospitalized for surveillance. During his hospitalization, he developed dyspnea, myalgias, arthralgias, fatigue, and persistent chest pain. Further studies showed elevation of cardiac enzymes (Troponin: 0.92 U/L (N: <0.5 U/L), creatine kinase (CK) 127 U/L (N: 48-100), CK-MB 25 U/L (N: 5-12 U/L), lactate dehydrogenase (LDH) 428 U/L (N: 100-250 U/L), aspartate aminotransferase (AST) 41 U/L (N: 5-30 U/L)), echocardiography reported pericarditis and left ventricular ejection fraction (LVEF) of 43%, MRI showed an LVEF of 40%. He was treated with ibuprofen 400 mg every eight hours and colchicine 1 mg every 24 hours for 14 days, without improvement. Given that he did not respond to nonsteroidal anti-inflammatory agents (NSAIDs) and severe chest pain persisted it was decided to give a pulse of methylprednisolone (1 g/day) followed by prednisone tapering dose. 

**Figure 2:**
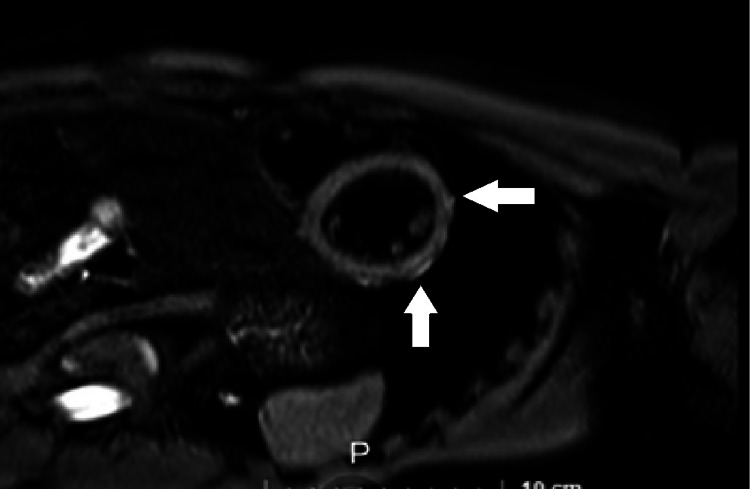
MRI T2-STIR Sequence (2020) Mild global myocardial hyperintensity is observed (arrows)

**Figure 3:**
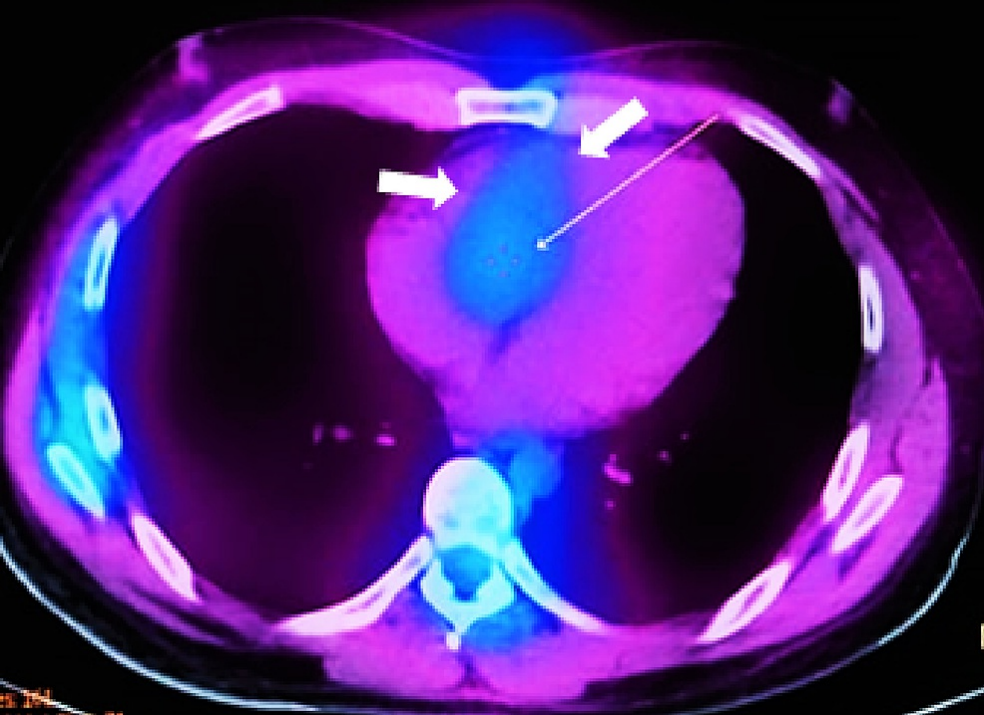
Cardiac Scintigraphy (August 2020) Zones of cold areas that show hyper inflammation (arrows)

Due to persistent cardiac manifestations (tachycardia, dyspnea, and fatigue), imaging studies were taken, including MRI and scintigraphy (Figures 2, 3), showing myopericarditis (Table 1). He is currently taking prednisone (5 mg/day) with no new symptoms. Imaging studies (echocardiography and MRI) were taken after one year, reporting normal LVEF (60%) and mild anteroseptal hypokinesia, LVEF: 60%, T2-STIR mild transmural hyperintensity in the anteroseptal region. Clinical manifestations have improved, but he still has evidence of alterations in imaging studies (Table 1). He received the first dose of COVID-19 vaccine in April 2021, the symptoms were not related to vaccination status. 

**Table 1:** Imaging studies Results and evolution of imaging studies in the follow up of one year. Improvement of LVEF based on Echocardiography and MRI (from 40% to 60%). Improvement of global hypokinesia to mild anteroseptal hypokinesia.* LVEF (Left Ventricular Ejection Fraction). MRI (Magnetic Resonance Imaging). STIR (Short T-l Inversion Recovery). PAH (Pulmonary Hypertension)*

Imaging studies	2020	October 2021
Echocardiography	Mild PAH (45 mmHg) LVEF: 40% Mild diastolic dysfunction	No PAH (21 mmHg) LVEF: 60% No diastolic dysfunction
Holter	Inappropriate sinus tachycardia and paroxysmal sinus tachycardia	It was not done
MRI Ga-67	Inferoseptal hypokinesia LVEF 40%. T2-STIR mild global myocardial hyperintensity	Mild anteroseptal hypokinesia LVEF: 60% T2-STIR mild transmural hyperintensity of the anteroseptal region
Cardiac Scintigraphy	Inflammatory process in the myocardium	It was not done

## Discussion

Since the beginning of the COVID-19 pandemic, many symptoms have been described related to the acute phase but also there are some reports that symptoms may appear or persist after weeks, which is known as long COVID syndrome. Recently, the World Health Organization issued the Delphi Consensus in which long COVID (post-COVID) is discussed, defining it as patients with a history of SARS-CoV-2 infection, three months from the initial phase of COVID-19 with symptoms new or persistent from the initial phase that last at least two months (fluctuating or relapsing), with impact on daily functioning and cannot be explained by another diagnosis [7]. The main risk factors for long COVID are being female, more than five symptoms and severe disease during the acute phase [6].

Myopericarditis diagnosis is given when there are two or more of the following: chest pain, pericardial rub, suggestive electrocardiography abnormalities, and pericardial effusion; however, when there is evidence of myocardial damage by elevation of cardiac enzymes or suggestive imaging, as in this patient with LVEF of 40%, it is called myopericarditis [8]. The etiology of myopericarditis has been related to viral infections mediated by cytotoxic or cytolytic effects causing inflammation, including SARS-CoV-2 [9], in the case of our patient we ruled out infections other than COVID-19, autoimmune and infiltrative causes. Many reports of myocarditis related to the acute phase of the disease have been reported; only one case of myopericarditis during the recovery phase of COVID-19 has been reported describing an elderly critically ill patient after six weeks of the acute phase. In addition, there are no known reports of myopericarditis related to long COVID syndrome in young patients [10].

This patient met the criteria for myopericarditis as a manifestation of long COVID syndrome. He had no comorbidities or risk factors. However, observations suggest that patients who develop mild symptoms at the beginning of the disease may also present long COVID syndrome [6]. Among the imaging studies of utility are transthoracic echocardiography (which detects ventricular dysfunction due to myocardial involvement and pericardial effusion), and cardiac MRI shows diffuse myocardial edema [8], cardiac scintigraphy was performed to rule out infiltrative disease in which cold areas of myocardial hypoperfusion were observed suggestive of myocarditis. However, it is emphasized that nuclear images should not be performed regularly since the availability is limited in most centers. In the case of myocarditis, a definitive diagnosis is made by myocardial biopsy [11], although, in patients with typical symptoms of myocarditis and imaging tests that guide the diagnosis, recent studies mention that biopsy can be avoided, so it is suggested to carry out the diagnosis based on imaging [12]. The patient was treated with NSAIDs and colchicine, according to the European guidelines for pericardial disease; perhaps, his evolution improved gradually after one year using second-line therapy (glucocorticoids [GC]) [8]. 

Based on the knowledge of cytokine storms, he was given methylprednisolone, which has been described as empiric treatment for giant cell, chronic, autoimmune, and eosinophilic myocarditis. This decision was made for the effects of GC on the synthesis inhibition of cytokines (IL-1, IL-2, IL-4, IL-5, IL-6, IL-12, IL-18, GM-CSF, TNF-α, IFN-γ). The described regimen has been described as a pulse dosing of methylprednisolone and then tapering with prednisone during four weeks, reducing 10 mg every two weeks until maintaining doses of 5-10 mg/day during one year according to the patient response [13,14].

## Conclusions

This young patient presented chest pain, dyspnea, and tachycardia after the acute phase of COVID-19 infection. He was diagnosed with myopericarditis with fluctuating symptoms and met the criteria for long COVID. This case highlights that a healthy patient who exhibits mild symptoms of COVID-19 may present long COVID. The cardiac imaging studies are of utility in the diagnosis of myopericarditis. GC is recommended to attenuate inflammation. In this patient, the manifestation of myopericarditis began after the acute phase of the disease, therefore we consider it as a post-COVID myopericarditis that has been persistent, and it is considered a case of long COVID syndrome according to the new nomenclature proposed by the World Health Organization. Since there are no cases reported of young patients with diagnosis of persistent myopericarditis as a presentation of long COVID syndrome, the prognosis is still unclear. Nevertheless, this patient has been responding to treatment with GC. 
